# Metastatic pilomatrix carcinoma treated with stereotactic body radiation therapy

**DOI:** 10.1016/j.jdcr.2024.02.032

**Published:** 2024-03-15

**Authors:** Rufus Banks, Jino Park, Linda Doan, Erin Healy, Jeremy P. Harris

**Affiliations:** a University of California–Irvine, Irvine, California; b Department of Radiation oncology, UC Irvine Medical Center, Orange, California; c Department of Dermatopathology, UC Irvine Medical Center, Orange, California

**Keywords:** metastatic pilomatrical carcinoma, metastatic pilomatrix carcinoma, pilomatrical carcinoma, pilomatrixoma, pilomatrix carcinoma, pilomatrix carcinoma and radiation, radiation oncology, radioresistance, stereotactic body radiation therapy

## Introduction

Pilomatrix carcinoma is a rare cutaneous adnexal neoplasm originating from hair follicles with propensity for recurrence and metastasis.^1^ Although standard treatment guidelines have not been established, surgical resection with wide margins is generally recommended to achieve local control. Mohs micrographic surgery is also utilized since these tumors frequently emerge in cosmetically sensitive areas like the head and neck region.^2^ However, in the case of nodal or distant metastases, the efficacy of chemotherapy and radiation remains limited and uncertain. Herein, we present a case of pilomatrix carcinoma with upfront nodal metastasis that had distant progression during the course of adjuvant radiation, and with no response to spine stereotactic body radiation therapy (SBRT).

## Case presentation

**Fig 1:**
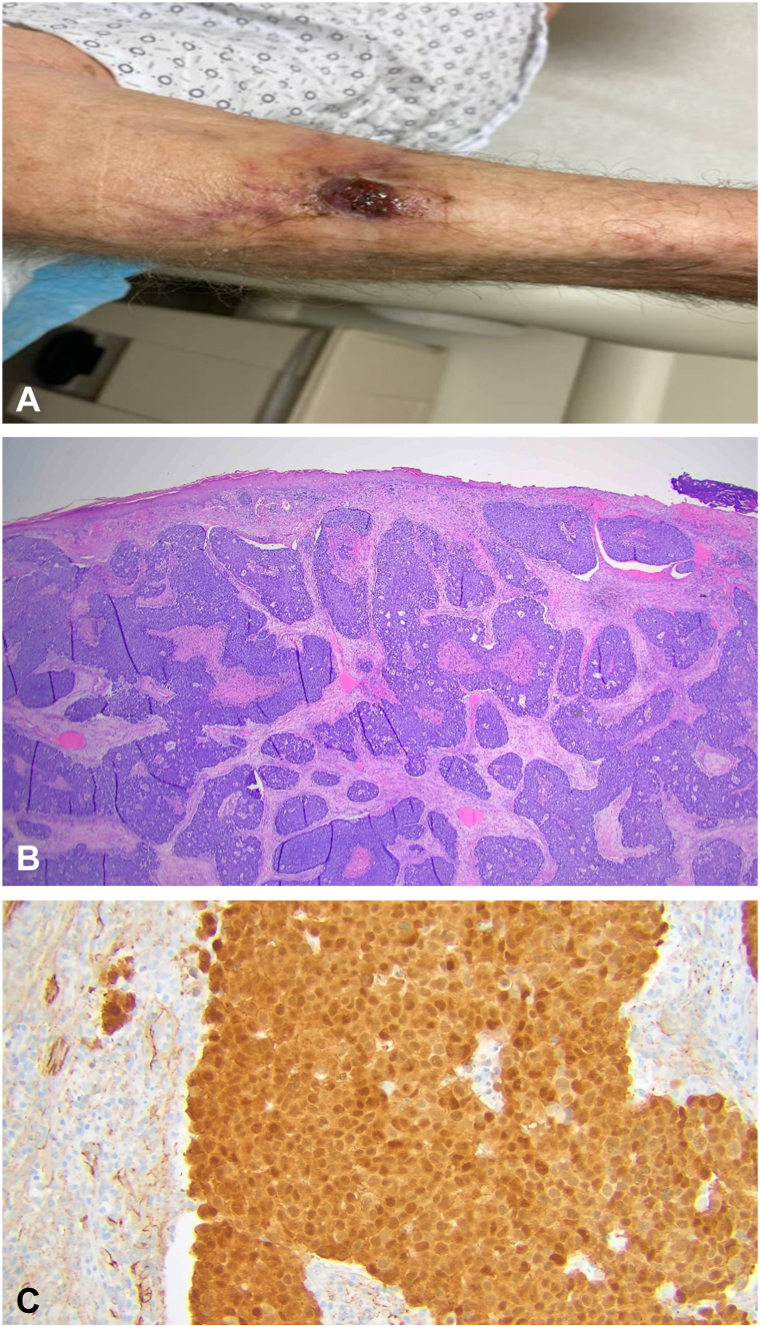
**A,** Recurrent pilomatrix carcinoma on patient’s left forearm. **B,** Low power view (20×) of resected specimen, H&E stain. **C,** High power view (200×) of specimen demonstrating nuclear B-catenin staining.

An 85-year-old male former smoker with a medical history of diabetes, hypertension, and interstitial lung disease presented to his dermatologist with an enlarging hyperpigmented mass on his left forearm. Shave biopsy demonstrated invasive basaloid carcinoma, and he was initially treated with Mohs micrographic surgery. Within 7 months, recurrence was noted arising within the center of the scar (Fig 1). Fluorodeoxyglucose positron emission tomography/CT revealed a hypermetabolic 1.8 cm lesion on the left forearm (4.7 SUV_max_) and a 1.3 cm left axillary node (5.6 SUV_max_), without evidence of distant metastases. Ultrasound-guided core needle biopsy of the lymph node confirmed carcinoma. Management included wide local excision and left axillary lymphadenectomy. Surgical specimen showed a 3.7 cm basaloid carcinoma staining positive for B-catenin with presence of shadow cells supporting a diagnosis of pilomatrix carcinoma (Fig 1). The closest margin was 0.7 cm (peripheral) and 0.3 cm (deep) on the primary specimen, and additional margins were excised in all directions to ensure negative margins were achieved on frozen section. Three out of 13 axillary lymph nodes exhibited metastatic foci, largest being 0.3 cm and without extranodal extension.

**Fig 2:**
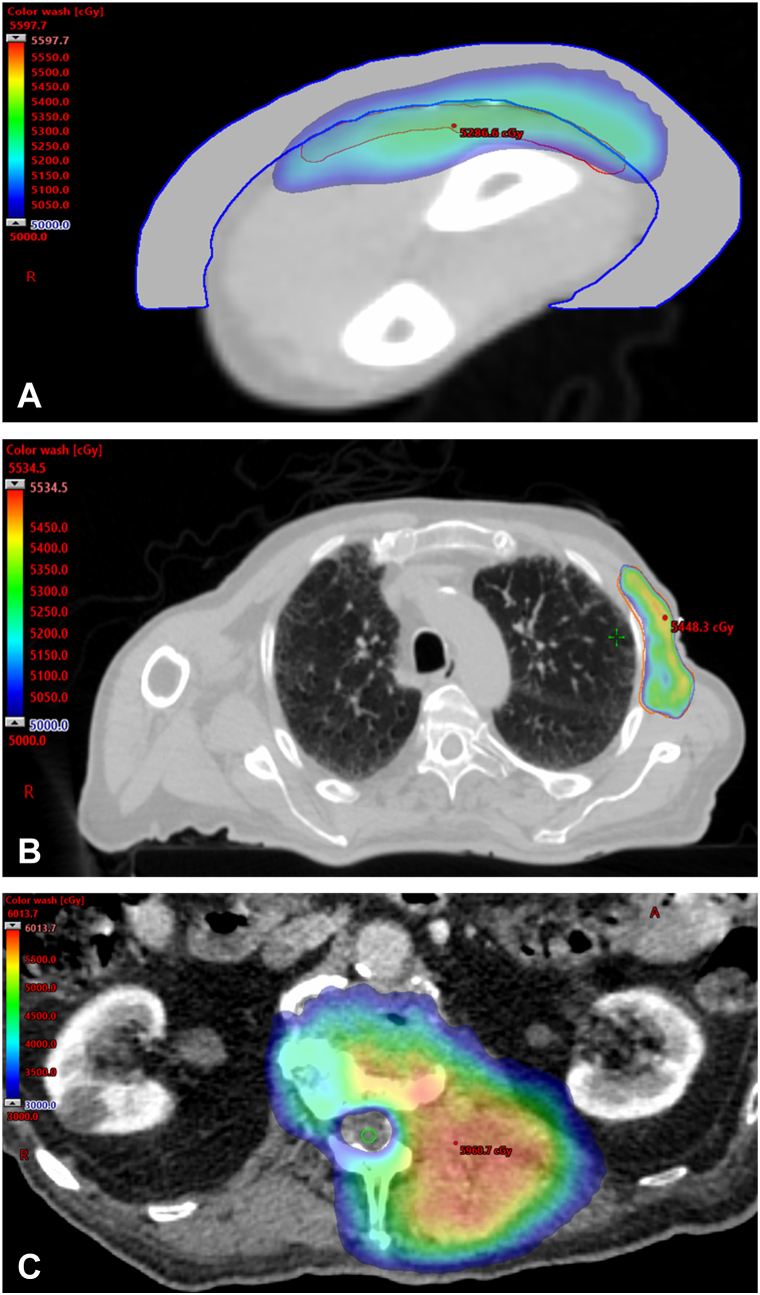
**A,** CT axial view of left forearm demonstrating en face electron radiation plan to a dose of 50 Gy. 1 cm bolus overlies the forearm in order to distribute dose near skin surface. **B,** CT axial view of chest showing highly conformal IMRT photon radiation plan for left axilla. **C,** CT axial view of lumbar spine demonstrating SBRT plan for L1 metastasis. Colorwash shows 30 Gy (75%) isodose line avoiding spinal cord (*green*). *CT*, Computed tomography; *IMRT*, intensity-modulated radiation therapy; *SBRT*, stereotactic body radiation therapy.

Six weeks after surgery, adjuvant radiation commenced. En face electrons (12 MeV) and intensity-modulated radiation therapy photon (6 MV) techniques were utilized to target the left forearm and axilla, respectively, delivering a dose of 50 Gy in 25 fractions to each site (Fig 2). However, during the course of the radiation, the patient developed worsening thoracolumbar pain prompting a magnetic resonance imaging, which revealed an L1 vertebral body lesion eroding bone and invading paraspinous musculature. There was no evidence of recurrence at the primary forearm site or axillary lymph nodes on physical exam or cone beam computed tomography. Biopsy of L1 confirmed metastasis, and radiation to the primary site and draining nodal region was terminated at 14 of 25 fractions.

**Fig 3:**
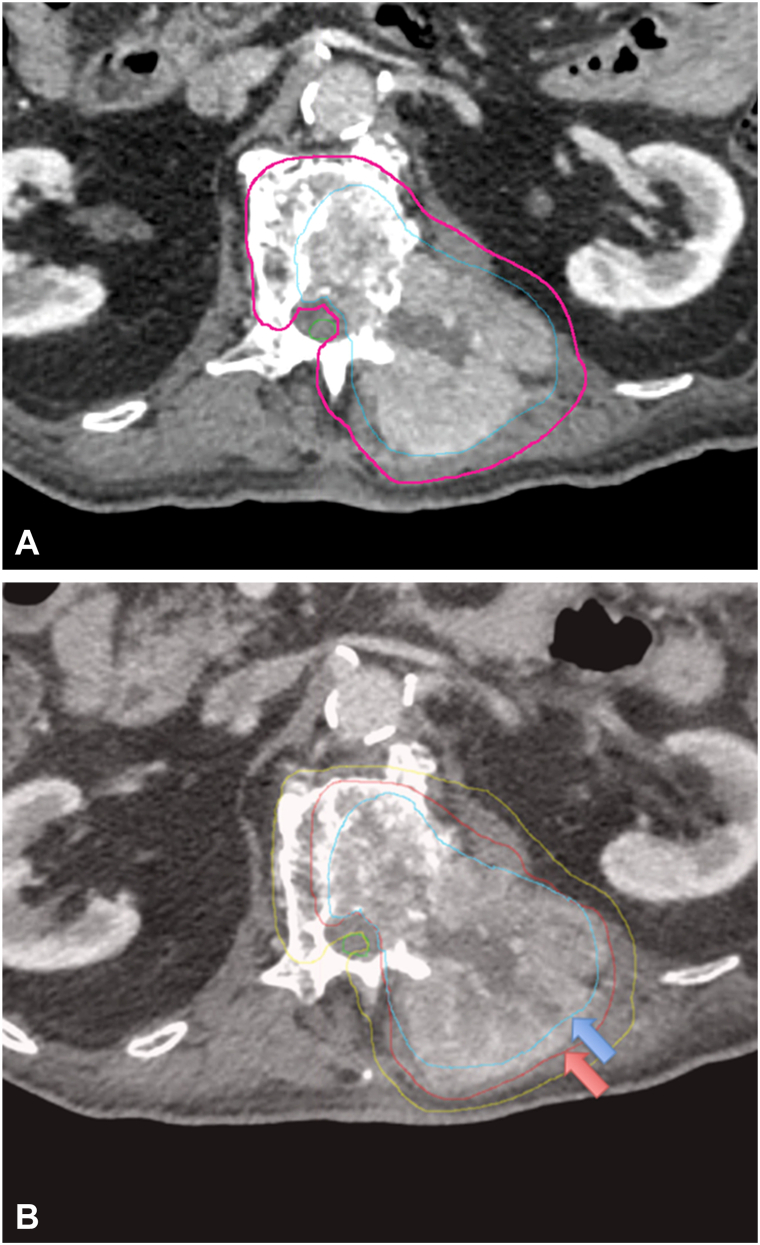
**A,** Initial SBRT plan delineating target volumes. *Blue* = GTV (gross tumor volume). *Pink* = PTV (planning target volume) which accounts for microscopic tumor spread and geometrical setup uncertainty. **B,** Adaptive SBRT plan highlighting tumor growth (*arrows*) in span of 3 weeks. *Blue* = initial GTV. *Red* = adaptive GTV. *Yellow* = adaptive PTV. *SBRT*, Stereotactic body radiation therapy.

**Fig 4:**
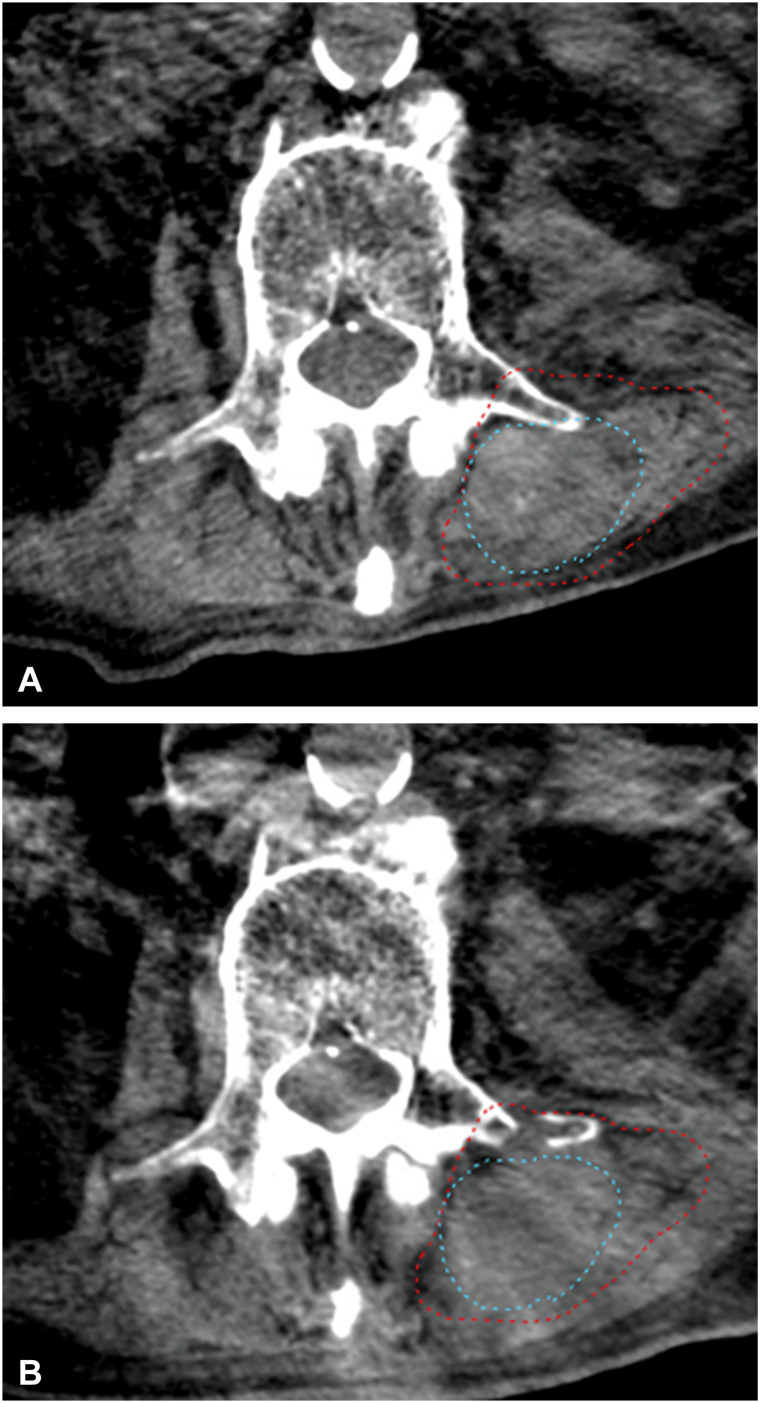
Axial cone beam CT of first (**A**) and fourth (**B**) day of SBRT. Disease progression is evident posteriorly where soft tissue density is extending beyond the adaptive GTV (*red*) and anteriorly where fracture of the left transverse process occured. *Blue* = initial GTV. *CT*, Computed tomography; *GTV*, gross tumor volume.

Attention was then shifted to the symptomatic spinal metastasis. After discussion of goals of care, the patient opted for palliative radiation with SBRT (10 MV) to a dose of 40 Gy in 5 fractions (Fig 2). He opted against chemotherapy following an informed discussion with his medical oncologist. Unfortunately, during the 3 weeks between the identification of metastasis and treatment initiation, there was rapid symptomatic and radiographic disease progression (Fig 3). Adaptive replanning occurred after 2 treatments to accommodate the growing lesion. Daily cone beam computed tomography imaging demonstrated disease progression and growth of the tumor through the SBRT course. By the fourth fraction, the soft tissue component of the tumor grew inferiorly into the transverse process of L2 resulting in fracture (Fig 4). Symptomatically, his pain worsened through the course of treatment and was managed medically with dexamethasone and multimodal analgesics including opioids. Ultimately, the patient completed only 4 of 5 fractions before opting for home hospice. He was recommended hospital evaluation for increased somnolence and altered mental status, but the family declined in line with the patient’s wishes. Further workup including an magnetic resonance imaging of brain was not performed. The patient died 2 days later at home, and specific cause of death was not reported.

## Discussion

Pilomatrix carcinoma presents a significant challenge in clinical practice due to its potential for both local and distant progression. Local recurrence rates after excision are high at 31% and occur early at an average of 6.5 months.^3^ Rates of metastasis are reported to be as high as 17%.^4^ The challenge is further compounded by resistance to chemotherapy.^5^ The case herein demonstrated a rapid in-field progression of metastatic pilomatrix carcinoma during radiation, reflecting the aggressive nature of this cutaneous malignancy and perhaps suggesting a component of radioresistance to even high doses of radiation delivered with SBRT. However, it should also be noted that radiation generally takes 2-3 weeks to take full effect, and this extreme case of gross tumor progression may not accurately illustrate the long-term efficacy of definitive or adjuvant radiation. Others have reported that radiation achieved good local control of pilomatrix carcinoma in the adjuvant setting.^3^

A similar case to the current report was published in 1999 by Bremnes et al, in which a thoracic spine metastasis was treated with a single posterior photon field to a palliative dose of 28 Gy in 7 fractions prescribed to a depth of 40 mm. The patient progressed, developed paraplegia during treatment, and died few days after treatment completion. The rapid progression of this patient was despite the receipt of cisplatin and 5-FU chemotherapy.^6^

The rarity of pilomatrix carcinoma made it challenging to gather sufficient data for a comprehensive understanding of its tumor biology, hindering the establishment of standardized therapies. Notably, mutations in *CTNNB1*, the gene encoding B-catenin, are characteristics of pilomatrix carcinoma. B-catenin is a downstream effector in the WNT pathway, which acts as a signal for proliferation and differentiation.^7^ Oncogenic mutations in CTNNB1 may offer clues for novel systemic therapies. Numerous clinical trials and preclinical evaluations are underway on WNT/B-catenin targeted therapies for various other solid tumors, and such agents may have a role for more effective treatment of pilomatrix carcinoma.^8^

## Conflicts of interest

None disclosed.
